# Influence of a proinflammatory state on postprandial outcomes in elderly subjects with a risk phenotype for cardiometabolic diseases

**DOI:** 10.1007/s00394-022-02870-7

**Published:** 2022-03-30

**Authors:** Yannik Bernd Schönknecht, Silke Crommen, Birgit Stoffel-Wagner, Martin Coenen, Rolf Fimmers, Peter Stehle, Sarah Egert

**Affiliations:** 1 Department of Nutrition and Food Science, Nutritional Physiology, University of Bonn, Nussallee 9, 53115 Bonn, Germany; 2 Institute of Clinical Chemistry and Clinical Pharmacology, University Hospital Bonn, Bonn, Germany; 3 Clinical Study Core Unit, Study Center Bonn, Institute of Clinical Chemistry and Clinical Pharmacology, University Hospital Bonn, Bonn, Germany; 4 Institute of Medical Biometry, Informatics and Epidemiology, University Hospital Bonn, Bonn, Germany

**Keywords:** Proinflammatory state, Interleukin-6, CVD-risk phenotype, Postprandial metabolism

## Abstract

**Purpose:**

Low-grade inflammation in obesity is associated with insulin resistance and other metabolic disturbances. In response to high-energy meal intake, blood concentrations of inflammatory markers, glucose and insulin rise. The aim of this study was to examine whether a basal inflammatory state influences postprandial responses.

**Methods:**

A randomized crossover trial was performed in 60 participants with a cardiometabolic risk phenotype (age 70 ± 5 years; BMI 30.9 ± 3.1 kg/m^2^). Each participant consumed three different iso-energetic meals (4300 kJ): a Western diet-like high-fat meal (WDHF), a Western diet-like high-carbohydrate meal (WDHC) and a Mediterranean diet-like meal (MED). Blood samples were collected when fasted and hourly for 5 h postprandially and analyzed for glucose, insulin, interleukin-1β (IL-1β), interleukin-6 (IL-6) and endothelial adhesion molecules. Based on fasting serum C-reactive protein (CRP) concentrations, participants were assigned to a high inflammation (CRP ≥ 2.0 mg/L; *n* = 30) or low inflammation (CRP < 2.0 mg/L; *n* = 30) group, and postprandial outcomes were compared.

**Results:**

Plasma IL-6, glucose and serum insulin increased after all meals, while IL-1β and endothelial adhesion molecules were unchanged. The high inflammation group had higher fasting and postprandial IL-6 concentrations than the low inflammation group, although the IL-6 response slope was similar between groups. In response to the WDHC meal, participants in the high inflammation group experienced a higher glycaemic response than those in the low inflammation group.

**Conclusion:**

A basal proinflammatory state results in higher absolute fasting and postprandial IL-6 concentrations, but the increase in IL-6 relative to basal levels is not different between high and low inflammation groups. Elevated glycaemic response in the high inflammation group may be due to inflammation-induced short-term insulin resistance.

The trial was registered at http://www.germanctr.de and http://www.drks.de under identifier DRKS00009861 (registration date, January 22, 2016).

## Introduction

Low-grade systemic inflammation (‘metaflammation’) is a characteristic of the obese state since adipose tissue releases a wide range of inflammatory mediators [1, 2]. The source of inflammatory mediators within adipose tissue is not fully elucidated: although adipocytes themselves play a role, infiltrating macrophages are also important [1]. Obese adults have higher circulating concentrations of inflammatory markers than lean adults (measured in the “resting state”, e.g., fasting), and these are believed to play a role in the development of insulin resistance and other metabolic disturbances of the metabolic syndrome and type 2 diabetes [1]. Blood concentrations of inflammatory markers are lowered following energy restriction and fat mass loss [1, 3]. In the hours following consumption of a high-energy meal, there is an elevation in circulating inflammatory mediator concentration, which is exaggerated in obese and type 2 diabetic subjects [1]. Evidence suggests that repeated exposure to postprandial inflammation stresses metabolic adaptation systems, leading to a dysmetabolic state [2]. In addition, the postprandial inflammation is closely associated with endothelial activation, indicated by the increased expression of endothelial adhesion molecules [4].

Recently, we reported that intake of energy-rich meals (~ 4300 kJ/meal) resulted in glycemia, lipemia and an inflammatory response in populations of older adults with a risk phenotype for cardiometabolic diseases [5, 6]. A meal typical of the Mediterranean diet resulted in favorable effects on glycemic, insulinemic and lipemic responses compared with two meals typical of Western dietary patterns [5]. However, as the inflammatory response was present to the same degree after each of the three iso-energetic meals, we suggested that energy intake is the main predictor of postprandial inflammation [5]. However, to the best of our knowledge, there are no studies investigating the influence of baseline inflammatory state on the inflammatory response to meal intake in older adults with metabolic syndrome traits. Thus, the aim of the present secondary analysis of data of our previous postprandial study [5] was to investigate whether a basal, low-grade inflammatory state would influence inflammatory responsiveness to the intake of different high-energetic meal patterns.

## Subjects and methods

### Subjects and study design

Participants were recruited in Bonn, Germany, via advertisements in local newspapers, public posting and flyers. Details of the study design and subject recruitment, enrollment and randomization have been described previously [5]. In brief, interested volunteers (*n* = 127) aged 60–80 years with a BMI > 27 kg/m^2^ attended screening, which included physical assessments, laboratory assessments, medical history and a dietary questionnaire. Overweight or obese participants were included if they had the following traits of metabolic syndrome: (1) visceral fat distribution (waist circumference ≥ 94 cm for men and ≥ 80 cm for women); (2) pre-hypertension (systolic BP ≥ 120–139 mmHg and/or diastolic BP ≥ 80–89 mmHg) or hypertension (systolic BP ≥ 140–159 mmHg and/or diastolic BP ≥ 90–99 mmHg); and (3) at least one of the following criteria: impaired glucose tolerance (fasting plasma glucose ≥ 5.55 mmol/L) and/or dyslipidemia (fasting serum triglycerides ≥ 1.7 mmol/L or serum HDL cholesterol < 1.0 mmol/L for men and < 1.3 mmol/L for women) and/or a proinflammatory state (high-sensitivity C-reactive protein ≥ 2.0 mg/dL) [5]. A total of 60 subjects (34 males, 26 females) were included in the study. All subjects completed the trial, and their respective data were included in the analysis. All study procedures were approved by the ethics committee of the Medical Faculty of the University of Bonn, Germany. Written informed consent was obtained from all subjects prior to inclusion. This trial was registered at http://www.germanctr.de and http://drks.de under identifier DRKS00009861 (registration date, January 22, 2016). The participants (*n* = 60) [5] were retrospectively divided into high inflammation and low inflammation groups and data were reanalysed.

The postprandial study was conducted as a randomized controlled crossover trial. Individuals participated in three, 5 h meal tests. Participants were assigned to the three different test meals by block randomization. Venous blood sampling was conducted prior to the test meal (0 h) and at 1, 2, 3, 4 and 5 h after finishing the test meal. Participants consumed an iso-energetic and iso-nitrogen test meal at each visit. The test meals were representative of real-life dietary intake: a Western diet-like high-fat (WDHF) meal (rich in total fat, SFA, and animal protein), a Western diet-like high-carbohydrate (WDHC) meal (rich in refined carbohydrates) and a Mediterranean diet-like (MED) meal (rich in unsaturated fatty acids, dietary fiber, and antioxidants). The energy and macronutrient composition of the meals are shown in Table 1, and detailed descriptions of meal composition are presented in Schönknecht et al. [5]. The test meals were consumed in the morning as the first meal of the day and had to be ingested within 20 min.

**Table 1:** Energy content and macronutrient composition of the test meals [adapted from [5]]

Energy/nutrient	MED meal	WDHF meal	WDHC meal
Energy, kJ	4238	4230	4241
Carbohydrates, g	133	94	145
Carbohydrates, EN %	53	37	58
Mono- and disaccharides, g	51	45	87
Protein, g	26	26	26
Protein, EN %	10	10	10
Total fat, g	40	59	34
Total fat, EN %	36	53	31
SFA, g	6	32	19
MUFA, g	24	20	11
PUFA, g	9	4	2

*EN %* energy percent, *MED* Mediterranean diet-like meal, *WDHC* Western diet-like high-carbohydrate meal, *WDHF* Western diet-like high-fat meal

### Blood sample processing and analysis

Details of the pre-analytical procedures of fasting and postprandial blood samples have been described previously [5]. Serum total cholesterol was measured using polychromatic endpoint measurement, whereas serum HDL cholesterol, LDL cholesterol and triglycerides were assessed using endpoint measurement with a Dimension Vista 1500 analyser (Siemens Healthcare Diagnostics). Plasma glucose was assessed via bichromatic endpoint measurement (Dimension Vista 1500 analyser), and serum insulin was determined using chemiluminescent-immunometric assay (Immulite 2000 analyzer, Siemens Healthcare Diagnostics). Serum CRP (high sensitive, hs) was determined using a turbidimetric immunoassay (Cobas 8000 modular analyser series). Hs-interleukin-1β (IL-1β) and hs-interleukin-6 (IL-6), soluble adhesion molecules E-selectin (sE-selectin), intercellular adhesion molecule-1 (sICAM-1) and vascular cell adhesion molecule-1 (sVCAM-1) were analyzed using commercially available enzyme-linked immunoassay kits (R&D Systems) [5].

### Statistical analysis

Based on CRP assessment at screening visit, participants were divided into (i) high inflammation (defined as fasting serum CRP ≥ 2 mg/L) and (ii) low inflammation (fasting CRP < 2 mg/L) groups [7, 8]. Postprandial responses were analyzed and compared between both groups using SPSS statistical software package (version 23; IBM). Proinflammatory state-related differences between the baseline characteristics at screening were analyzed using unpaired Student’s *t* tests. Statistical significance was defined as *P* < 0.05. Prior to analyses, the glucose, insulin and hs-CRP data were log-transformed.

Postprandial outcomes were tested for effects of inflammatory state, time and test meal by performing linear mixed models. Fasting values were included as covariates, and participants were included as random factors. When significant interactions were absent (*P* > 0.05), mixed model calculations were repeated without the interaction terms. Differences between fasting variables were tested using a mixed model analysis. Residuals obtained from the calculations were inspected for normality to control for the fit of the statistical model. Logarithmic transformation was applied before analysis if the residuals were not normally distributed, which was true for glucose, insulin, IL-6, sICAM-1 and sVCAM-1 data.

The incremental AUC (iAUC) was calculated for the concentration of all postprandial parameters as a summary variable. Calculations were performed as reported previously [5]. Data are expressed as mean ± SEM, and all *P* values listed are two tailed.

## Results

### Baseline characteristics

Baseline characteristics are shown in Table 2. Of the 60 participants studied, 30 were assigned to the high inflammation group (10 males, 20 females), and 30 were assigned to the low inflammation group (24 males, 6 females). Age, BMI, waist circumference, fat mass, systolic blood pressure, glucose, insulin and serum lipids were comparable between groups. Significantly higher diastolic blood pressure was evident in the high inflammation group compared with the low inflammation group (*P* = 0.033).

**Table 2:** Baseline characteristics of participants for the high inflammation and low inflammation group^a^

Parameter	High inflammation group	Low inflammation group	*P* value
N (male/female)	30 (10/20)	30 (24/6)	–
Age (years)	70.4 ± 5.8	69.2 ± 4.9	0.402
BMI (kg/m^2^)	31.0 ± 3.3	30.9 ± 3.0	0.918
Waist circumference (cm)	104.9 ± 9.5	106.4 ± 7.0	0.237
Fat mass (%)	37. 0 ± 6.6	30.8 ± 7.0	0.273
Systolic BP (mmHg)	149 ± 17	147 ± 15	0.578
Diastolic BP (mmHg)	91 ± 9	87 ± 8	0.033
Plasma glucose (mmol/L)	5.34 ± 1.06	5.56 ± 0.93	0.310
Serum insulin (pmol/L)	75.6 ± 31.4	89.7 ± 37.9	0.119
Serum triglycerides (mmol/L)	1.80 ± 0.73	2.03 ± 0.95	0.306
Serum total cholesterol (mmol/L)	5.43 ± 0.99	5.24 ± 0.92	0.428
Serum HDL cholesterol (mmol/L)	1.48 ± 0.43	1.37 ± 0.29	0.250
Serum LDL cholesterol (mmol/L)	3.37 ± 0.81	3.25 ± 0.79	0.568
Serum hs-CRP (mg/L)	7.17 ± 4.56	0.99 ± 0.48	< 0.001

Values are means ± SD. Comparisons were performed using the unpaired Student’s *t* test

*BP* blood pressure, *hs-CRP* high-sensitivity C-reactive protein

^a^High inflammation group, CRP ≥ 2.0 mg/L; Low inflammation group, CRP < 2.0 mg/L

### Postprandial response of IL-6, IL-1β and endothelial adhesion molecules

**Fig. 1:**
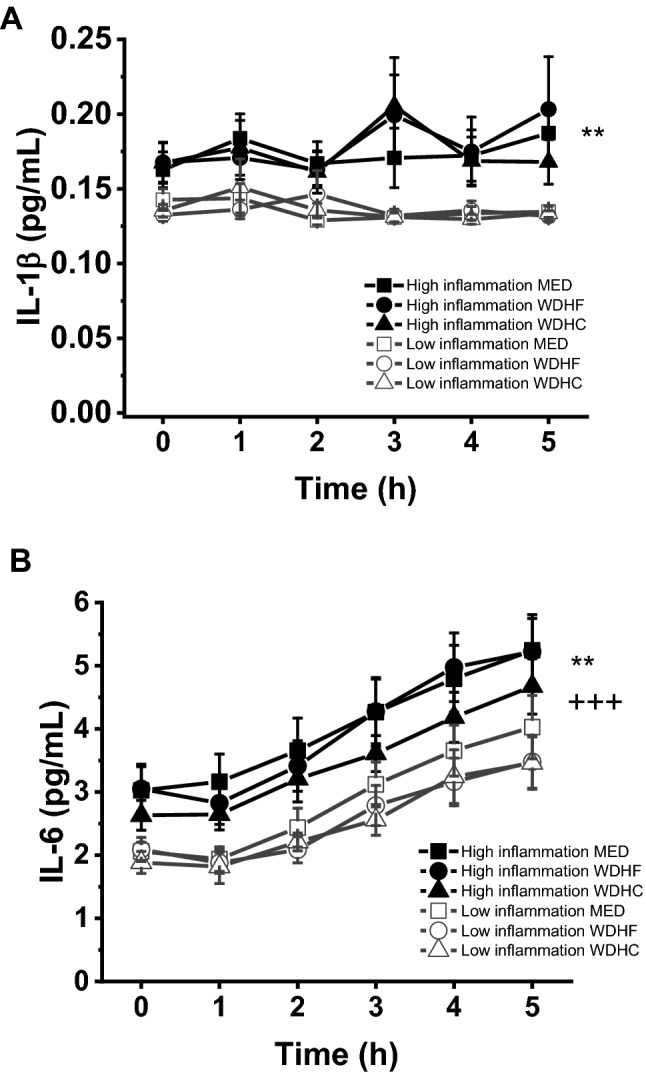
Fasting and postprandial plasma concentrations of IL-1β (A) and IL-6 (B) for high and low inflammation groups. +  +  + *P* < 0.001 for time effect. *P < 0.05; **P < 0.01 for group effect. IL-1β; interleukin-1β; IL-6, interleukin-6; MED, Mediterranean diet-like meal; WDHC, Western diet-like high-carbohydrate meal; WDHF, Western diet-like high-fat meal

A proinflammatory state had a significant effect on inflammatory responses to test meal intake (Fig. 1). Participants from the high inflammation group showed higher fasting and postprandial IL-6 concentrations than the low inflammation group (*P* = 0.001). However, increases in IL-6 concentration over the postprandial period were similar between the high and low inflammation groups: in both groups, plasma IL-6 increased by approximately 100% (low inflammation group, 103%; high inflammation group, 100%) from baseline in response to meal intake (Fig. 1).

Fasting and postprandial concentrations of IL-1β were significantly higher in the high inflammation group than in the low inflammation group (*P* = 0.005). However, concentrations of IL-1β did not significantly change over time. No differences in fasting or postprandial concentrations of sICAM-1, sVCAM-1 and sE-selectin between the high and low inflammation groups were observed; endothelial adhesion molecules also did not significantly change over time (data not shown). Summary variables (iAUC) for IL-6, IL-1β and endothelial adhesion molecules were not affected by inflammatory status (Table 3).

**Table 3:** iAUC values for postprandial glucose, insulin, IL-1β, IL-6 and endothelial adhesion molecules

Parameter	High inflammation group			Low inflammation group			*P* value inflammation × meal	*P* value inflammation effect	*P* value meal effect
	MED	WDHF	WDHC	MED	WDHF	WDHC			
Glucose iAUC (h*mmol/L)	4.8 ± 1.2	3.6 ± 0.6	6.5 ± 1.3	3.8 ± 0.8	2.9 ± 0.5	3.9 ± 0.61	0.735	0.096	0.016
Insulin iAUC (h*pmol/L)	1556 ± 159	1447 ± 127	2095 ± 229	1884 ± 266	1405 ± 139	2238 ± 275	0.344	0.577	< 0.001
IL-1β iAUC (h*pg/mL)	0.04 ± 0.03	0.03 ± 0.04	0.03 ± 0.04	− 0.03 ± 0.02	0.01 ± 0.02	0.01 ± 0.01	0.480	0.197	0.759
IL-6 iAUC (h*pg/mL)	14.9 ± 3.1	9.1 ± 3.1	11.4 ± 2.8	13.5 ± 3.0	7.4 ± 2.2	9.6 ± 2.9	0.912	0.645	0.269
sE-Selectin iAUC (h*ng/mL)	− 1.2 ± 1.6	− 7.2 ± 1.5	− 5.9 ± 2.1	− 4.1 ± 1.5	− 3.9 ± 1.7	− 5.7 ± 2.4	0.238	0.174	0.884
sICAM-1 iAUC (h*ng/mL)	− 11.7 ± 10.9	− 21.3 ± 16.6	2.4 ± 15.5	5.0 ± 12.0	− 14.0 ± 15.8	− 29.6 ± 21.1	0.261	0.818	0.450
sVCAM-1 iAUC (h*ng/mL)	− 86.7 ± 38.4	− 104.4 ± 47.4	− 133.2 ± 43.5	− 75.1 ± 34.1	− 31.2 ± 43.2	− 52.5 ± 39.8	0.668	0.098	0.827

Values given as mean ± SEM

*MED* Mediterranean diet-like meal, *IL-1β* interleukin-1β, *IL-6* interleukin-6, *sE-selectin* soluble E-selectin, *sICAM-1* soluble intercellular adhesionmolecule-1, *sVCAM-1* soluble vascular cell adhesionmolecule-1, *WDHC* Western diet-like high-carbohydrate meal, *WDHF* Western diet-like high-fat meal

### Postprandial responses of glucose and insulin

**Fig. 2:**
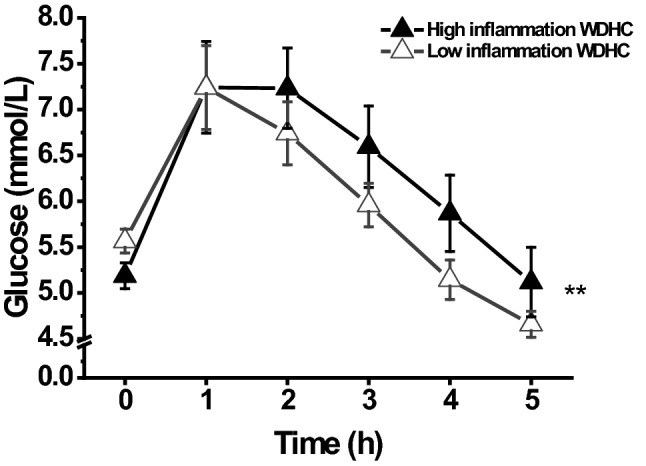
Fasting and postprandial plasma glucose concentration after Western diet-like high-carbohydrate (WDHC) meal intake in high and low inflammation groups. **P < 0.01 for differences between high and low inflammation groups

The inflammatory state had a meal-dependent effect on glycaemic responses: the high inflammation group had significantly higher postprandial glucose concentrations 2–5 h after the WDHC meal than the low inflammation group (*P* = 0.006) (Fig. 2). Glucose iAUC values also tended to be higher in the high inflammation group than in the low inflammation group (*P* = 0.096) (Table 3). Glucose responses to the MED and WDHF meals were not affected by inflammatory state (kinetic data not shown). No differences were observed in insulin responses between the high and low inflammation groups (data not shown).

## Discussion

The present report is a secondary analysis using data from the Diet–Body–Brain (DietBB) postprandial study conducted in 60 older individuals with a cluster of metabolic syndrome traits [5]. We found that the relative magnitude of the inflammatory response to meal intake, measured by changes in plasma IL-6 concentrations over a 5-h period, was similar between subjects with and without proinflammatory markers. In addition, glucose response to a WDHC meal was higher and more prolonged in participants in a proinflammatory state.

Our data indicate that relative postprandial inflammatory response intensity is not related to fasting inflammatory status in overweight/obese subjects with metabolic syndrome traits. The current literature on the impact of meals on inflammatory outcomes according to basal inflammatory status is extremely limited. Phillips et al. [9] assessed the effect of a high-fat meal on markers of inflammation in (i) lean, (ii) obese non-diabetic and (iii) type 2 diabetic men. In accordance with our data, obese non-diabetic men presented an inflammatory state, indicated by higher fasting hs-CRP concentrations than lean men (0.2 vs. 1.3 mg/L). Absolute concentrations of IL-6 following the high-fat meal increased in all groups, but there were no differences in response slopes relative to fasting values between groups. In contrast to our study, baseline IL-6 concentrations did not differ between groups [9]; however, the study was limited to males, baseline characteristics (e.g., age, BMI, body composition) differed between groups, and the sample size was small (*n* = 10 per group).

Another study compared postprandial vascular, inflammatory and leukocyte adherence responses between lean and obese men in an inflammatory state after high-fat milkshakes [10]. No differences in postprandial responses between lean and obese participants were observed. Likewise, Schwander et al. [11] challenged 19 normal-weight and 18 obese men with 2100, 4300 and 6300 kJ high-fat meals. Fasting concentrations of CRP (0.9 ± 0.9 vs. 3.0 ± 3.0 mg/L), but not IL-6, differed between groups. None of the energy doses led to the identification of a significant difference in the postprandial response of inflammatory markers between normal-weight and obese participants. These data confirm that the higher absolute postprandial IL-6 concentrations we observed resulted from an overweight/obesity-induced basal inflammatory state, rather than an amplified inflammatory response. The functions and mechanism of action of IL-6 are complex, meaning that elevated IL-6 concentrations in the basal state do not necessarily result in an augmented postprandial inflammatory response. In addition, the metabolic effects of IL-6 depend on the cellular environment and the concentration [12].

Interleukin-6 is the most frequently assessed marker of inflammation in the postprandial period and consistently increases between 4 and 8 h after intake of a high-fat meal [13]. Elevated IL-6 concentrations have been linked to multiple clinical parameters (e.g., blood pressure, insulin sensitivity and coronary artery disease) [13]. Furthermore, proinflammatory cytokines, such as IL-6 and tumor necrosis factor-α, have been implicated in the progression of atherosclerosis [14] and type 2 diabetes [15, 16]. Although more research is needed regarding the clinical relevance of short-term IL-6 fluctuations after meal intake, one may speculate that over the course of a day, accumulation of IL-6 resulting from repeated meal intake may trigger metabolic dysmetabolism, atherosclerosis and diabetes progression.

Our data also indicate that basal inflammatory state influences postprandial glucose response. Participants from the high inflammation group had higher glucose concentrations over the postprandial period than the low inflammation group. Interestingly, this finding was only observed after WDHC meal intake, not after WDHF and MED meal intake, indicating that the inflammation-related impairment of the glycaemic response may be associated with the amount of carbohydrate consumed. These group differences may be reasoned by the association of low-grade inflammation with insulin resistance [17–19]. The subjects in the high-inflammatory group show elevated concentrations of inflammation markers (hs-CRP and IL-6), with the consequence of postprandially impaired insulin sensitivity and subsequently decreased glucose uptake. The repeated exposure to postprandial increased glycemia represents the contribution of low-grade inflammation to insulin resistance.

One mechanism potentially linking the higher glycaemic response in participants from the high inflammation group is the interference by cytokines in insulin action. Proinflammatory signaling molecules activate certain inflammatory pathways, which interfere with intracellular insulin-receptor signaling by activating serine kinases, directly blocking insulin action [1, 2, 18]. Chronically elevated IL-6 concentrations in the basal inflammatory state are associated with substantial metabolic effects, e.g., systemic insulin resistance [19]. In vitro and in vivo models show that insulin action is inhibited in adipocytes, hepatocytes and endothelial cells exposed to cytokines (IL-1β, IL-6 and tumor necrosis factor-α) [20] and other proinflammatory mediators [21–23].

To the best of our knowledge, the current study is the first to report that, relative to baseline levels, inflammatory responses to meal intake are similar between subjects with and without a proinflammatory phenotype. One strength of our study is that we focused on a clinically relevant population, i.e., overweight/obese older individuals, and that we used a whole-diet approach to study acute effects of meals on several cardiometabolic parameters [5]. In addition, retrospective division of the 60 participants into high and low inflammation groups based on baseline CRP concentrations resulted in evenly distributed (*n* = 30 per group) groups with similar anthropometric and metabolic characteristics. Furthermore, assessment over 5 h is adequate to cover most postprandial responses, and parameters were measured hourly, allowing in-depth evaluation of postprandial kinetics.

One limitation of the present work is that it is an exploratory, and thus secondary, data analysis. The study was originally designed to investigate the effects of three different meal patterns on postprandial lipemic, glycemic and inflammatory responses; thus, the presented data are ancillary examinations. In addition, we only selected plasma/serum markers (cytokines and adhesion markers) to characterize the inflammatory response. Currently, no consensus exists regarding inflammatory mediators that best represent low-grade inflammation [1, 24]. In addition, we are far from a consensus regarding response features (i.e., timing and magnitude) after a meal of even the most commonly assessed markers of inflammation [13]. Future postprandial studies should include further biomarkers, such as the examination of immune cell-activation of inflammatory gene expression profiles or inflammatory omic responses [24]. Also, further biomarkers, such as galectin-3, may be considered in postprandial studies to examine the relation between low-grade inflammation and insulin resistance [19]

In conclusion, our data indicate that individuals in a proinflammatory state have higher absolute cytokine blood concentrations postprandially than individuals without elevated proinflammatory markers. The magnitude of the inflammatory response relative to baseline levels was similar between groups, demonstrated by IL-6 concentrations increasing equally for individuals in the high and low inflammation groups. In addition, the glucose response to a WDHC meal was higher and more prolonged in participants in a proinflammatory state. These data suggest that low-grade inflammation may contribute to short-term insulin signaling impairment during the postprandial period, thereby leading to augmented postprandial glucose responses.

## Abbreviations

BP: Blood pressure
hs-CRP: High-sensitivity C-reactive protein
EN %: Energy percent
iAUC: Incremental area under the curve
IL-1β: Interleukin-1β
IL-6: Interleukin-6
MED: Mediterranean diet-like meal
sE-selectin: Soluble E-selectin
sICAM-1: Soluble intercellular adhesion molecule-1
sVCAM-1: Soluble vascular cell adhesion molecule-1
WDHF: Western diet-like high-fat meal
WDHC: Western diet-like high-carbohydrate meal
